# Advanced Sensing and Delivery Technologies for Nose-to-Brain Administration: From Nanocarriers to Sensor-Integrated Organ-on-Chips

**DOI:** 10.3390/s26082523

**Published:** 2026-04-19

**Authors:** Xiaoxue Liu, Ruoqi Chen, Fan Wu, Bingqian Yu, Guojin Zhou, Sunhong Hu, Hongjian Zhang, Ping Wang, Boyang Xu, Liujing Zhuang

**Affiliations:** 1 Biosensor National Special Laboratory, Key Laboratory for Biomedical Engineering of Education Ministry, Zhejiang Key Laboratory of Intelligent Sensing Technology and Advanced Medical Instrument and Key Laboratory for Biomedical Engineering of Ministry of Education, College of Biomedical Engineering & Instrument Science, Zhejiang University, Hangzhou 310027, China; liuxiaoxue@zju.edu.cn (X.L.); cnpwang@zju.edu.cn (P.W.); 2 Zhejiang Key Laboratory of Drug Prevention and Control Technology, National Narcotics Laboratory Zhejiang Regional Center, Hangzhou 310053, Chinaxbyxby2012@163.com (B.X.); 3 The MOE Frontier Science Center for Brain Science & Brain-Machine Integration, The State Key Laboratory of Brain-Machine Intelligence, Zhejiang University, Hangzhou 310027, China; 4 Cancer Center, Binjiang Institute of Zhejiang University, Hangzhou 310027, China

**Keywords:** nasal drug delivery, nose-to-brain pathway, nanoparticles, microneedles, organ-on-a-chip

## Abstract

Central nervous system (CNS) disorders represent a growing healthcare burden, and various drugs are developed for their treatment. However, the blood–brain barrier (BBB) prevents over 98% of therapeutics from reaching brain tissue. Intranasal delivery provides a promising alternative by exploiting olfactory and trigeminal nerve pathways to circumvent the BBB. This review surveys recent advances in nose-to-brain delivery technologies, from carrier design to evaluation methods. Polymeric and lipid-based nanocarriers show enhanced mucosal penetration and prolonged residence time, and microneedle platforms further enable controlled drug release with minimal discomfort. To evaluate these delivery strategies, sensor-integrated organ-on-chip models provide more physiologically relevant testing than static cultures. Although persistent challenges such as rapid mucociliary clearance and formulation stability remain, combining nanotechnology with microfluidic devices and computational modeling shows potential for developing patient-specific therapeutics.

## 1. Introduction

The accelerating aging of the global population has escalated neurological diseases into a critical healthcare burden [1]. Central nervous system (CNS) disorders, including Alzheimer’s disease (AD), Parkinson’s disease (PD), and multiple sclerosis, are a major class of neurological diseases. Despite this growing burden, therapeutic interventions for CNS disorders remain historically limited and costly. For example, primary brain tumors are still treated mainly with surgical resection, systemic chemotherapy, and radiation, and effective alternatives remain limited [2]. A major hurdle in developing alternatives is the blood–brain barrier (BBB), which is formed by specialized brain endothelial cells and acts as a barrier to many solutes. This restriction is enforced by endothelial tight junctions, membrane transporters, and vesicular mechanisms [3]. Almost all macromolecular drugs and more than 98% of small-molecule drugs cannot cross the BBB, with only molecules under 400 Da or those with high lipid solubility able to pass through via passive diffusion [4,5]. Due to the highly selective and protective barrier, the effect of conventional pharmacotherapy for CNS disorders is limited [6].

To bypass the barrier, non-invasive strategies have been developed to improve CNS drug delivery. One of the promising strategies is intranasal (IN) administration [7]. Due to the unique anatomy of the nasal cavity and the presence of the olfactory and trigeminal nerves, intranasal administration can provide direct CNS access and partially bypass the BBB [8,9]. The short distance between the nasal cavity and the brain provides drug delivery with an advantage of less degradation and higher efficiency. Intranasal therapy dates back to Ayurvedic “Nasya karma” and gained renewed scientific interest as a systemic delivery strategy in the early 1980s [10]. Currently, there is growing interest in nose-to-brain delivery strategies. Various formulation approaches have been developed to optimize nasal drug delivery, including the utilization of mucoadhesive polymers (e.g., cellulose derivatives, polyacrylates, starch, and chitosan), sol–gel techniques, and pH-dependent absorption systems to overcome multifaceted delivery challenges [11]. These formulation advances have enabled CNS drug delivery, enhanced diagnostics, and integrated theranostic platforms for improved brain targeting [12].

**Figure 1:**
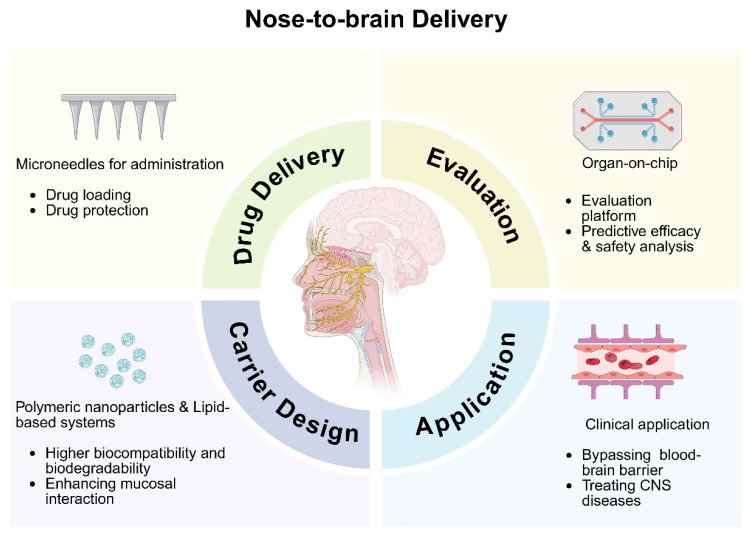
Schematic illustration of the integrated nose-to-brain delivery strategy. Created in https://BioRender.com.

Despite these promising developments, intranasal drug delivery faces several challenges, including rapid mucociliary clearance, enzymatic degradation, and limited residence time in the nasal cavity [13,14]. To overcome these limitations, innovative approaches are being actively explored across multiple technological fronts, e.g., novel nasal sprays, nanotechnology-based drug carriers and hydrogel-based systems. This review highlights recent advances in nanocarriers, microneedle platforms, and organ-on-chip evaluation for nose-to-brain delivery, with an emphasis on translational potential (Figure 1). We also discuss the current challenges in nose-to-brain drug delivery application and suggest future directions in this field.

## 2. The Principles of Nose-to-Brain Drug Delivery

The nose serves as a primary chemical organ for humans to perceive the world. As a potential route for drug delivery, it offers three main transport mechanisms: olfactory pathway, trigeminal nerve pathway and respiratory pathway.

The external nose is supported by a framework of bone and hyaline cartilage, covered by skin and muscle tissue. The upper portion is bony and rigid, while the lower portion is cartilaginous and mobile. Anatomically, the human nasal cavity is a complex physiological structure with a total volume of approximately 15 mL and an overall surface area of 150 cm^2^ [15]. Functionally, the nose is divided into four distinct sections: the nasal vestibule, atrium, respiratory region, and olfactory region. Among these, the nasal vestibule occupies a minor area of about 0.6 cm^2^; while the olfactory region—which serves as the primary portal for direct nose-to-brain delivery—is located at the roof of the cavity and covers only about 10% of the total nasal surface area [16].

### 2.1. The Blood–Brain Barrier

**Figure 2:**
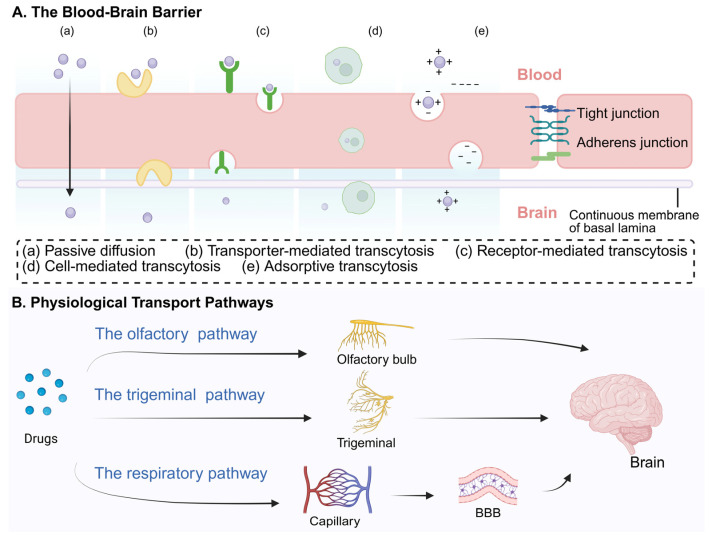
Blood–brain barrier transport mechanisms and physiological nose-to-brain pathways. (**A**) Various transcytotic mechanisms exist for molecules to traverse the BBB endothelium. (**B**) Following intranasal administration, drugs can enter the brain via the olfactory and trigeminal pathways (bypassing the BBB) or enter the systemic circulation via the systemic pathway (or the respiratory pathway). Created in https://BioRender.com.

The blood–brain barrier (BBB) is the primary obstacle to CNS drug delivery. As a key component of the neurovascular unit, it tightly regulates solute exchange between the blood and brain [17]. Unlike peripheral capillaries, the BBB endothelium presents especially low permeability due to the absence of fenestrae, and low rates of transcytosis. For macromolecules and most polar solutes, the BBB’s low paracellular permeability is primarily maintained by tight junctions (TJs) that are between adjacent brain endothelial cells. Tight junctions are composed of proteins such as claudins, occludin, and zonula occludens-1, which effectively seal the paracellular pathway [18], as shown in Figure 2. This highly selective barrier blocks nearly 98% of small-molecule drugs and almost all biologics from entering the brain [19].

Beyond physical barriers, the BBB also employs active efflux mechanisms. The endothelium utilizes ATP-binding cassette transporters to actively efflux xenobiotics and drugs back into the bloodstream. Furthermore, specialized transporters including MFSD2a (the major facilitator transporter for essential omega-3 fatty acids), have been identified to suppress transcytosis in CNS endothelial cells [20,21,22]. Despite advances, much research on BBB still remains incomplete due to the complexity of the CNS. These physiological blockades limit the efficacy of traditional systemic administration for CNS therapy, hence necessitating alternative routes, such as nose-to-brain delivery, to bypass the BBB.

### 2.2. The Olfactory Pathway

The olfactory pathway represents a critical route for direct nose-to-brain delivery, which can circumvent the BBB. After intranasal administration, therapeutic modalities first reach the olfactory mucosa and can traverse the cribriform plate via nerve bundles. From the olfactory nerves, these drugs can enter the cerebrospinal fluid and the olfactory bulb, facilitating their distribution into the brain [16,23].

This transport occurs via two distinct mechanisms: the intraneuronal pathway and the extraneuronal pathway. The intraneuronal pathway involves axonal transport, a process that is relatively slow and may require hours to days [21]. In contrast, the extraneuronal pathway uses perineural channels for rapid transport, enabling therapeutics to reach the brain within minutes [22]. For instance, Frey et al. [24]. demonstrated that ^125^I-labeled nerve growth factor rapidly appeared in the olfactory bulb, cerebrum, and brainstem following intranasal administration, supporting a direct extracellular pathway to the brain. These mechanisms enable the olfactory pathway to deliver drugs not only to the olfactory bulb but also to deeper brain regions such as the cortex, hippocampus, and amygdala [25].

### 2.3. The Trigeminal Pathway

The trigeminal pathway constitutes a supplementary route for direct nose-to-brain drug delivery. The trigeminal nerve, the largest cranial nerve, comprises three branches: the ophthalmic (V1), maxillary (V2), and mandibular (V3). The ophthalmic and maxillary branches are critical for intranasal delivery because they directly innervate the nasal mucosa. Specifically, the ophthalmic branch innervates the dorsal part of the nasal mucosa and the anterior nose, whereas the maxillary branch supplies the turbinates [8]. The trigeminal pathway connects the nasal passages to the brainstem and spinal cord. After crossing the mucosa, therapeutics are transported along trigeminal nerve fibers and enter the brainstem primarily at the level of the pons [26]. From there, drugs are distributed caudally to the medulla and the rest of the hindbrain, and rostrally toward the midbrain and diencephalon. Additionally, the ethmoidal branch of the trigeminal nerve passes through the cribriform plate, providing a distinct route to the forebrain [26,27].

Transport via the trigeminal pathway occurs through both intracellular and extracellular mechanisms. Multiple studies have validated rapid delivery of various therapeutic agents via this route. For instance, Thorne [26] reported that insulin-like growth factor-I (IGF-I) rapidly reached the brain via the trigeminal pathway following intranasal administration. Similarly, other agents such as lidocaine and Interferon-β-1b have been shown to utilize this axonal route for CNS entry [27,28]. Ross et al. [28] found that at similar blood concentrations, intravenous administration of Interferon-β-1b yielded 88–98% lower CNS levels compared to intranasal delivery, confirming the pathway’s superior efficiency in bypassing the BBB. Furthermore, Kyrkanides et al. [29] demonstrated that even large payloads, such as viral vectors for gene therapy, can undergo retrograde axonal transport via the trigeminal system to treat neurodegenerative conditions. These findings highlight the versatility of the trigeminal pathway for delivering a diverse array of therapeutics.

### 2.4. The Respiratory Pathway

The drugs deposited in the nasal cavity can also reach the brain through the blood circulation. The respiratory pathway, also referred to as the systemic pathway, starts with the respiratory region of the nasal cavity. The respiratory region of the nasal cavity comprises the superior, middle and inferior turbinates, and possesses the largest surface area. It is innervated by the ophthalmic and maxillary branches of the trigeminal nerve [30]. Functionally, drug entry into the blood circulation through this route is supported by two key features: the mixture of continuous and fenestrated endothelium in this region that enables absorption of small and large molecules; an extensive vascular network within the respiratory epithelium. The respiratory mucosa contains more blood vessels than the olfactory region, thus facilitating drug absorption into systemic circulation [31]. The transport mechanism occurs in a two-phase process. First, drugs deposited in the respiratory region, particularly near the inferior turbinate, undergo absorption into the bloodstream [32]. Second, counter-current exchange mechanisms deliver drugs to the carotid arterial blood supply, which serves the brain and spinal cord [16]. This pathway demonstrates molecular selectivity, showing preferential transport for small lipophilic molecules while being less efficient for high-molecular-weight and hydrophilic compounds [33]. Since drugs enter the systemic circulation, they are once again subject to the restriction of the BBB and systemic clearance, similar to oral or intravenous administration. As a result, the respiratory pathway is generally not considered the primary mechanism for direct brain targeting.

## 3. Advances in Intranasal Drug Delivery

### 3.1. Nanocarrier-Based Systems

Nasal drug delivery has evolved from optimizing aerosol deposition for respiratory treatment to engineering nanocarriers for direct central nervous system (CNS) targeting. Traditional nasal sprays primarily rely on inertial impaction for deposition in the anterior nasal cavity [34]. To reach the brain, particles should bypass the respiratory region and deposit in the olfactory slit. However, standard sprays often fail because of the complex nasal geometry.

**Figure 3:**
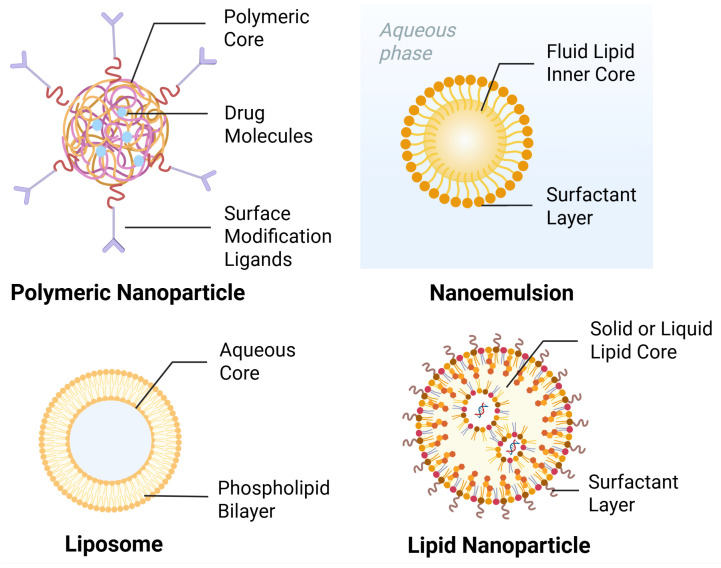
Schematic structures of representative nanocarriers for intranasal drug delivery. Created in https://BioRender.com.

Although the anatomical pathways provide the route, effective delivery requires overcoming biological barriers, such as mucociliary clearance and enzymatic degradation. Substantial work has focused on engineering nanocarriers that protect payloads and enhance mucosal interaction. Currently, polymeric nanoparticles and lipid-based systems represent the most validated technologies when carrying drugs. Representative nanocarrier structures are shown in Figure 3.

#### 3.1.1. Polymeric Nanocarriers

Polymeric nanoparticles are biocompatible carriers typically ranging in size from 10 to 1000 nm [35]. Poly (lactic-co-glycolic acid) (PLGA) nanoparticles are extensively studied due to their high biocompatibility, biodegradability, and robust physical properties [36]. Unmodified PLGA, however, suffers from rapid clearance, necessitating targeted surface functionalization strategies.

Lectins can target specific moieties on the olfactory epithelium to enhance neuronal uptake. Gao et al. [37] reported that lectin-functionalized nanoparticles using wheat germ agglutinin (WGA) achieved a 1.8-fold increase in brain uptake of model fluorescent markers compared to unmodified nanoparticles, primarily via adsorptive endocytosis. Safety evaluation using both in vitro toad palate and in vivo rat nasal mucosa models demonstrated negligible nasal ciliotoxicity after repeated administration for 6 days, with ciliary movement duration (12 h) comparable to negative controls (12.25 h). Ahmad [38] demonstrated that trans-activator of transcription (TAT) peptide (derived from HIV-1) functionalized mPEG-PDLLA micelles enhanced cellular uptake and brain delivery compared to unmodified ones. In parallel, three key principles can enhance paracellular transport: strong mucoadhesion that prolongs nasal residence, electrostatic surface interactions that invert particle charge to strengthen mucus adhesion, and transient opening of epithelial tight junctions to facilitate paracellular transport [39]. Recent experimental validation has demonstrated these principles in practice. Barros [40] found that chitosan-coated liposomes exhibited a 1.7-fold increase in ex vivo mucoadhesion, achieved a strong positive zeta potential (+60.8 ± 6.6 mV) for enhanced electrostatic interactions with negatively charged mucus, and showed 1.3-fold enhanced permeation through nasal epithelium. Intranasal administration delivered 48.2 ± 8.8% of the ghrelin dose to the brain within 25 min, whereas free ghrelin was undetectable. These mechanisms extend residence time, promote epithelial traversal, and reduce mucociliary clearance, offering a framework for optimizing brain delivery across polymeric systems. Surface charge significantly influences nanoparticle safety, with cationic polymeric nanoparticles potentially inducing platelet aggregation and hemolysis, whereas anionic nanoparticles demonstrate reduced toxicity profiles [41]. However, current studies remain at the proof-of-concept stage, and there is still limited information on the precise brain regions reached after administration. Because many of these systems rely on various functionalisation or coating strategies to enhance nasal uptake, and testing conditions are not standardized, direct comparison across studies remains difficult [42]. The long-term safety of surface-modified polymeric nanoparticles, including possible mucosal toxicity, neurotoxicity, and the immunogenicity of certain coating materials, still requires further evaluation [43].

#### 3.1.2. Lipid-Based Systems

While polymeric nanocarriers offer versatile surface functionalization, lipid-based systems provide superior biocompatibility and low toxicity risks associated with synthetic polymers and organic solvents. Lipid-based carriers, including emulsions, liposomes, solid lipid nanoparticles (SLNs) and nanostructured lipid carriers (NLCs), are capable of encapsulating lipophilic drugs and facilitating transport across the lipid-rich BBB [16]. SLNs comprise biocompatible lipids that remain solid at room and body temperatures. NLCs are often regarded as second-generation SLNs, which incorporate solid and liquid lipids [44]. NLCs offer improved drug encapsulation and sustained release, whereas SLNs demonstrate controlled drug release and increased bioavailability [45]. Recent studies have investigated agomelatine-loaded SLNs to treat depression. Fatouh et al. [46] showed that intranasal administration of these optimum SLNs had a direct transport percentage of 47.37 and a drug-targeting efficiency of 190.02, which indicated a more successful brain delivery than the intravenous route. This work also suggests that lipid carriers can protect drugs from hepatic first-pass metabolism while maximizing nose-to-brain transport [47]. Beyond agomelatine, fluoxetine-loaded lipid nanoparticles are also used in depression treatment. For example, Vitorino et al. [48] developed fluoxetine-loaded nanostructured lipid carriers, and the resulting nanoparticles exhibited a particle size of 154 nm, with encapsulation efficiency and drug loading of approximately 74% and 13%, respectively. This method manifests enhanced intranasal brain delivery and antidepressant effects in behavioral models. The effects of formulation parameters on SLN performance are not always predictable, and variables expected to accelerate drug release may produce unexpected outcomes, highlighting the complexity of formulation optimization [46].

### 3.2. Microneedles for Drug Delivery

Though nanocarriers offer a promising vehicle for drug delivery, researchers continue exploring administration routes that maximize therapeutic efficacy. In recent years, interest has gradually shifted from parenteral and oral routes towards topical therapies, such as the transdermal drug delivery system [49], which reduces the overall dose needed to reach targeted site. The microneedle drug delivery system has gained popularity as it can overcome the skin’s barrier properties and deliver drugs into the circulatory system. Microneedle-mediated transdermal delivery using nanocarriers is also a common strategy in drug delivery because this combination overcomes the barrier of the stratum corneum, and protects drugs from elimination in skin tissues at the same time [50].

#### 3.2.1. Current Microneedle Fabrication Technologies

Microneedles are micron-scale structures that serve as a painless alternative to traditional injections for transdermal drug delivery. These drug delivery systems have attracted widespread attention due to their non-invasiveness, painless administration, and ability to overcome the barrier function of the stratum corneum [51].

Microneedle fabrication employs various techniques. Three-dimensional (3D) printing enables the production of high-quality microneedles with tunable characteristics using diverse materials. In addition, integration of artificial intelligence may further enhance precision [52]. For scalable production, micromolding and casting methods utilize silicon-based master molds and polymer replication processes. This process involves several steps: (1) 3D printing of a resin microneedle model with stable shape, (2) creating a polydimethylsiloxane (PDMS) intermediate mold to obtain the negative template, (3) pouring of liquid polymer precursors into the mold cavities, (4) thermal or UV curing [53]. Lithography and etching techniques provide high-resolution patterning capabilities, while laser ablation methods offer precise micromachining for complex geometries. Microneedles can be fabricated from various materials, including biodegradable polymers (PLA, PLGA, PCL), hydrogel-based systems with controlled swelling behavior, and hybrid composite materials for enhanced mechanical properties [51]. Recent progress has been made in bio-inspired designs. For instance, the blue-ringed octopus-inspired microneedle patches provide robust tissue surface adhesion through negative pressure and covalent/hydrogen bonding [54].

Each fabrication technology presents distinct advantages and limitations. Three-dimensional printing technologies offer design flexibility and rapid prototyping capabilities, making them ideal for personalized microneedle designs. However, their resolution limitations (typically > 100 µm) may restrict fabrication of ultra-fine needle tips required for certain applications [55]. By contrast, micromolding and casting methods provide excellent reproducibility and scalability for mass production, with superior surface quality and dimensional accuracy. However, they require significant initial investment in master mold fabrication and are less adaptable to design modifications. These micro-fabrication techniques enable applications in transdermal drug delivery, wound healing, tissue engineering, and biosensing. Microneedles address limitations of traditional drug delivery methods (including oral pills, nasal sprays, and intravenous injections) by providing controlled, localized, and patient-compliant therapeutic delivery.

**Figure 4:**
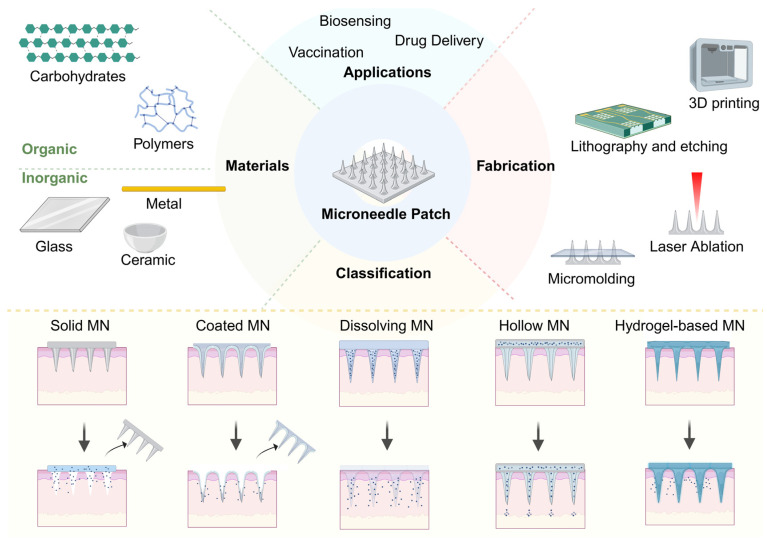
Schematic overview of microneedle systems. The diagram illustrates the fabrication techniques, constituent materials, and the classification of microneedles based on their drug delivery mechanisms. Created in https://BioRender.com.

Generally, microneedles can be classified into five categories, i.e., solid, drug-coated, dissolvable, hollow and hydrogel-based microneedles, according to microneedle-based devices (Figure 4). Solid microneedles, which are typically made of metal or silicon, are less convenient for patients because they require a two-step procedure. Drug-coated microneedles are always limited by the insufficient amount of drugs they carry [56]. This review thus focuses on dissolving, hollow, and hydrogel-forming microneedles, which show promise for nasal drug delivery.

#### 3.2.2. Dissolving Microneedles

Microneedles typically range from 50 to 900 μm in length, penetrating only the stratum corneum or superficial mucosal layers without reaching pain receptors. In this way, they offer a better choice for pain-sensitive individuals. Dissolving microneedles (DMNs) are a promising approach for drug delivery to the brain [57]. DMNs are micrometer-sized needles made of biocompatible matrices that dissolve in water, eliminating concerns related to residual sharps. Recent studies have explored drug-loaded DMNs for treating neurodegenerative diseases, including AD and PD, because of their ability to deliver drugs efficiently to the brain via multiple routes [58]. DMNs also offer various advantages, including biocompatibility, not leaving biohazardous waste, avoidance of first-pass metabolism, high cargo-loading capability, and controlled drug release [59,60]. More importantly, intranasal or transdermal delivery of drugs using DMNs can bypass the BBB and presystemic metabolism, providing therapeutic efficacy in the brain and enhancing patient compliance. These benefits must be balanced against matrix-dependent constraints on drug loading, insertion strength, and formulation robustness. Intracranial administration may be cumbersome and could compromise patient compliance, while transdermal application still causes mild local reactions such as swelling and erythema [61]. In some animal models, intranasal delivery triggers sneezing and subsequent drug loss, which remains a challenge that requires resolution.

Telmisartan (TMN), an angiotensin receptor blocker, has been formulated into nanocrystal-incorporated dissolving microneedles for brain-targeted delivery to treat Alzheimer’s disease. Madani et al. [62] reported that the nanocrystal formulation showed enhancement in drug release, reaching 89.51 ± 7.52% (2-fold higher than pure TMN). The pharmacokinetic parameters were improved, demonstrating enhanced bioavailability and effective brain delivery without skin irritation. In the context of neuro-oncology, glioblastoma multiforme (GBM), the most prevalent and fatal brain tumor with an annual incidence of 3.19 new cases per 100,000 people in the US, presents significant treatment challenges despite standard approaches (surgical resection, radiotherapy, and chemotherapy) [63,64]. To address this issue, a novel silk microneedle patch has been developed for on-demand multidrug delivery directly to the brain in GBM models. Silk protein can be extracted from natural silk cocoons and can be mixed with other additives such as nanoparticles or enzymes, to form functional silk-inks for inkjet printing [65]. The biocompatible silk matrix enables controlled delivery of three therapeutic agents: thrombin for hemostasis [66], temozolomide to induce DNA damage and apoptosis in tumor cells [67], and bevacizumab for anti-angiogenesis [68]. The patch circumvents blood–brain barrier limitations while maintaining drug stability (97% temozolomide activity retention versus 47% loss without silk protection) [69]. This system demonstrates decreased tumor volume and increased survival rates in mouse models, offering a controllable approach for clinical brain tumor treatment when conventional methods are insufficient. These studies highlight the potential of microneedle technology for brain-targeted delivery.

#### 3.2.3. Hollow Microneedles

In contrast to early types of microneedles, hollow microneedles (HoMNs) feature an internal cavity through which drugs are delivered under pressure, like conventional injections. HoMNs are particularly suitable for delivering high-viscosity or high-dose drugs [56]. They are usually designed to be compatible with applicators that enable precise and controlled drug delivery. Rather than relying on passive diffusion or dissolution, HoMNs require external pressure mechanisms to actively push liquids through the hollow channels [70]. Diverse applicators have been invented to optimize injection efficiency and delivery precision, including syringe-based manual systems and microfluidic devices [71]. HoMN delivery mechanism shows strong potential in transdermal drug delivery mainly for two reasons. First, it enables flexible drug delivery depth, ranging from the dermis to the subcutaneous and muscle layers. Delivery depth is a critical parameter affecting the absorption rate and must be carefully considered for effective delivery [72]. Second, HoMNs facilitate the reuse of approved drugs and offer considerable merits in drug development, saving time and resources.

HoMNs show clinical efficacy across multiple therapeutic applications with precise dosage control. For analgesic applications, fentanyl transdermal systems achieved effective pain management with dosages of 25–100 mcg through commercial patches like Duragesic^®^ [73], while lidocaine/epinephrine combinations delivered 7 mg/kg for local anesthesia [74,75]. Moreover, insulin delivery systems with glucose monitoring and automatic delivery mechanisms could revolutionize daily health management for diabetic patients [76]. The flexible dosage control makes HoMNs particularly suitable for chronic disease management.

#### 3.2.4. Hydrogel-Based Microneedles

Hydrogel-based microneedles (HMNs) comprise crosslinked polymers through chemical or physical processes, showing unique properties and application potential [77]. Dry HMNs possess sufficient mechanical strength to penetrate skin and absorb interstitial fluid upon insertion, forming continuous and clear channels for drug delivery. In their hydrated state, they maintain resilience, enabling the microneedles to be completely removed from the skin without leaving residues [78]. Crosslinking degree in the polymer’s three-dimensional network determines the swelling properties of HMNs, which allows for accurate control of drug-release rates and loading capacity. Compared to other types of microneedles, HMNs feature excellent biocompatibility, high loading capacity and tunable drug-release rates [79,80].

Since hydrogel enables sustained drug release, HMNs show broad applicability for treating various skin disorders, including alopecia, psoriasis, vitiligo and infectious diseases [81]. For instance, Li et al. [82] developed a PDA-JAKi microneedle delivery system fabricated by incorporating the janus kinase inhibitor tofacitinib into antioxidant polydopamine nanoparticles, which enhanced therapeutic efficacy in vitiligo treatment. Drug release and permeation in HMNs are primarily influenced by the properties of the polymers used, with different materials providing unique functions. Recent advances in HMN design, including the development of smart and multifunctional microneedles, have expanded applications to cancer, cardiovascular diseases, and chronic wounds. However, they still face challenges including poor mechanical properties and insufficient stability [83,84]. These limitations require continued research to overcome technical barriers and enhance clinical translation.

### 3.3. Specialized Delivery Modalities

#### 3.3.1. Hydrogel-Based Systems

Hydrogels are effective delivery platforms with mucoadhesive properties that extend drug residence time [85]. These three-dimensional crosslinked polymer networks can absorb water while maintaining structural integrity, making them ideal for sustained release. Thermosensitive hydrogels undergo sol–gel transition at body temperature, improving patient compliance by enabling liquid administration that gels in situ [86]. Peppas and colleagues found that hydrogel formulations could enhance bioavailability of therapeutic agents through controlled release [87]. Mucoadhesive hydrogel systems, particularly those containing chitosan or carbopol polymers, enable prolonged contact with nasal epithelium and increase drug absorption. These systems have successfully delivered neuropeptides like oxytocin for psychiatric disorders and growth factors for neuroprotection [85,88].

Temperature-sensitive hydrogels have achieved progress in nose-to-brain drug delivery. For example, Xu et al. [89] developed a self-assembled intranasal thermosensitive in situ hydrogel to co-deliver berberine (BBR) and evodiamine (EVO). In their study, the delivery system showed good release properties and antidepressant effects by regulating monoamine neurotransmitter metabolism, providing a noninvasive treatment strategy for the clinical treatment of depression. Teng et al. [90] created a nasal temperature-sensitive hydrogel containing edaravone and borneol inclusion complex (EDA-BP TSGS) that undergoes phase transition at physiological temperature, prolonging nasal residence time and enhancing bioavailability. Tested on rats, this system improved neurological deficits and reduced cerebral infarct areas, offering hope for safe and effective brain-targeted therapy in ischemic stroke treatment. Beyond passive encapsulation, Ryu et al. [91] employed a conjugation-based approach for delivering nucleic acid-protein therapeutics to treat stroke. They conjugated β-hydroxybutyrate to polyethylene glycol (PEG)-modified nanoparticles to enable monocarboxylate transporter 1-mediated brain delivery of CRISPR systems. This active targeting strategy achieved neuroprotective effects and improved motor function in stroke models. However, conventional chitosan/β-glycerophosphate (CS/B-GP) thermosensitive hydrogels may suffer from poor mechanical integrity, which enhances drug retention and lead to premature release or washout [92]. Consequently, by incorporating different nanomaterials, hydrogels are poised to play an increasingly vital role in clinical nasal drug delivery.

#### 3.3.2. Vaccine Applications

Nasal vaccination induces both systemic and mucosal immunity with direct CNS access. This dual immune response offers advantages over traditional parenteral routes by establishing protective immunity at mucosal surfaces where many pathogens first encounter hosts [93]. This approach shows promise for preventing viral encephalitis and developing vaccines targeting amyloid-beta and tau proteins in Alzheimer’s disease [94].

The nasal-associated lymphoid tissue (NALT) plays crucial roles in immune responses, serving as the primary site for nasal immune responses. NALT contains antigen-presenting cells that can efficiently process and present antigens to naive T and B lymphocytes [95]. In addition, unlike Peyer’s patches in the intestine, NALT shows unique characteristics in antigen uptake and immune activation.

Nanoparticle-based vaccines protect antigens from degradation and facilitate immune cell uptake [96]. These systems can enhance antigen stability, improve cellular uptake, and provide sustained antigen release for prolonged immune stimulation. Vaccine mucosal delivery technologies based on nanoparticles (such as lipid, polymeric and inorganic nanoparticles), provide stability and controlled release while enhancing mucosal adhesion [97]. Lipid nanoparticles offer high encapsulation efficiency, low toxicity, and enhanced cellular uptake. Chitosan nanoparticles can adhere to negatively charged mucoproteins, reducing nasal clearance [98], while gold nanoparticles act as antigen delivery scaffold. Conjugation-based strategies represent a promising alternative to enhance vaccine immunogenicity through active targeting mechanisms. Hartwell et al. [99] conjugated the receptor-binding domain (RBD) of severe acute respiratory syndrome coronavirus 2 (SARS-CoV-2) spike protein to amphiphilic lipids, enabling FcRn-mediated transmucosal uptake following intranasal administration. This lipid-conjugated RBD vaccine induced robust neutralizing antibody titers and amplified mucosal germinal center responses. However, some carriers, including PLGA nanoparticles, cannot undergo sterile filtration and show incomplete antigen release [100]. Materials such as chitosan require additional modification to improve biocompatibility and water solubility, increasing manufacturing complexity and cost [101]. Overall, these innovations support intranasal immunization as an efficient mucosal vaccine delivery method with a wider range of applications.

## 4. Organ-on-a-Chip

Advanced nanocarriers and microneedles are representative strategies for drug loading and delivery via the nose-to-brain route. However, before these formulations can be successfully translated to clinical practice, they must undergo rigorous preclinical evaluation. Traditional in vitro models rely upon static conditions and therefore lack essential microenvironmental factors such as shear stress and proper cell–cell interactions. To overcome these static limitations, organ-on-a-chip (OoC) technology represents an advanced in vitro modeling approach that more accurately recapitulates the dynamic in vivo human microenvironment [102]. Furthermore, integrated microsensors for real-time monitoring of both culture conditions and cell metabolism provide unprecedented insight into these complex systems.

Recent studies demonstrate that the monitoring of metabolic parameters and culture conditions with embedded sensors can provide many advantages in terms of information content, quality of drug screening experiments, and fundamental metabolic characteristics of tissues [103]. Sensors for different parameters such as oxygen [104], nitric oxide [105], pH [106], transepithelial electrical resistance (TEER) [107], glucose [108] and other biochemically significant analytes have been developed to characterize static and dynamic as well as 2D and 3D in vitro models.

### 4.1. Nasal-Related Organ-on-Chip Models

In the field of nasal drug delivery, researchers seek to establish optimal in vitro methods for evaluating the transepithelial transport and mucosal permeation of nasal formulations. Conventionally, this procedure is performed by administering drug solutions or suspensions onto compartmentalized donor-acceptor setups [109]. For example, Transwell^®^ or Snapwell inserts usually serve as the primary donor-acceptor cell culture platforms. On these platforms, nasal mucosa cell layer models are established using air-liquid interface (ALI) culture of nasal epithelial cells to investigate the therapeutic effect and delivery efficacy of IN drugs [110]. Nevertheless, these methods fall short in mimicking the realistic nasal drug administration. In the example above, Pozzoli et al. [111] integrated Snapwell inserts containing ALI-cultured nasal epithelial cells into a spherical expansion chamber that meets US Food and Drug Administration (FDA) requirements for nasal aerosol delivery testing. Although this approach enables direct exposure of cells to aerosolized nasal formulations within the chamber, it remains limited by static cell culture conditions, conventional drug testing protocols, and standard post-deposition analytical methods.

**Figure 5:**
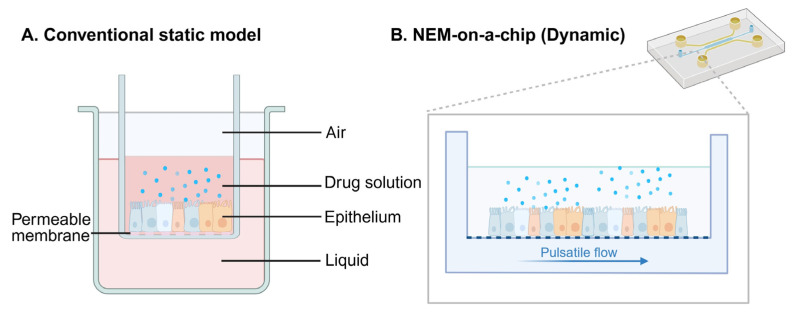
Schematics of (**A**) the conventional static model and (**B**) the dynamic NEM-on-a-chip device. Created in https://BioRender.com.

To address these limitations, organ-on-chip technology with embedded sensors offers a robust approach for nasal drug delivery research. It provides real-time insights into the metabolic state of the nasal mucosa. Unlike traditional methods, these microfluidic systems simulate the complex microenvironments and physiological functions of human organs, and therefore play a vital role in modern biomedical engineering [112]. They combine tissue engineering and microfabrication techniques to provide precise control over cellular microenvironments and recreate physiologically relevant conditions, thereby enabling more accurate in vitro models for drug testing and disease modeling [113]. Despite the growing interest in intranasal drug delivery, there is a lack of new technologies to accurately test the safety and efficacy of intranasal products. Previously, a human nasal epithelium mucosa (NEM)-on-a-chip was developed to support the ALI culture of RPMI 2650 nasal epithelial cells and to mimic the nasal mucus production and barrier function [114]. In this system, nasal drug transport was evaluated under either static or dynamic fluidic conditions in the acceptor channel, as shown in Figure 5. However, the donor channel could only be used for the administration of liquids or suspensions. In summary, existing microfluidic aerosol delivery systems still lack living tissue integration and adjustable airflow rates, both of which are necessary for realistic respiratory drug testing conditions [115]. Many organ-on-chip platforms primarily concentrate on drug solutions, while actual intranasal drug products typically exist as aerosols, powders, or gels [110].

From a sensing perspective, a key innovation lies in the integration of functionalized microelectrodes. Gholizadeh et al. [114] reported a human NEM-on-a-chip with a novel carbon nanofiber-modified carbon electrode to enable real-time, quantitative monitoring of intranasal drug delivery across an epithelial barrier. On this kind of chip, a Manostat Carter 12/6 cassette pump system (Barnant Co., Barrington, IL, USA) was used to apply a continuous circulating pulsatile flow of 3 mL phosphate buffer (PB, pH 7) through the acceptor channel at a flow rate of 0.5 mL/min. This setup enabled continuous and real-time quantification of drug transport across the nasal epithelium. Electrochemical analysis offers numerous advantages such as being reliable, time-efficient and requiring small sample volumes [116]. Therefore, this novel NEM-on-a-chip provides a low-cost and time-efficient alternative to the costly laborious conventional techniques for in vitro nasal drug transport assays. To further enhance the analytical capabilities, recent research has focused on integrating multimodal microsensors.

While sensor integration improves the functional readouts, researchers have also sought to replicate the structural complexity of the nasal cavity. Attempts also have been made to manufacture anatomically relevant 3D replicas of human nasal cavities for in vitro intranasal drug testing. The transparent nasal model is an anatomically relevant human nasal cavity model that facilitates the study of intranasal aerosol performance and qualitative assessment of drug deposition within the nasal cavity [115]. Researchers are continuously striving to establish a new immortalized nasal epithelial cell line as the basis for an improved 3D nasal mucosa cell culture model. For instance, Koch et al. [117] developed test platforms based on tissue-compatible microfluidic chips using immortalized porcine nasal epithelial cell lines, thereby establishing a dynamic artificial nasal mucosa (AnaMuc) on-chip model. Compared to the static AnaMuc model, the cell layer cultured under dynamic on-chip conditions demonstrates organotypic barrier properties like those of the nasal mucosa.

The evolution from conventional Transwell inserts to NEM-on-a-chip platforms with integrated sensors, dynamic flow, and aerosol delivery capabilities represents a paradigm shift in nasal drug delivery research. Current organ-on-chip models have successfully incorporated electrochemical sensing for real-time drug monitoring, TEER measurements for barrier integrity assessment, and dynamic flow conditions mimicking physiological shear stress.

### 4.2. Nasal Drug Evaluation

In vitro assessment of nasal aerosol delivery has been carried out in an OoC platform to study epithelial drug transport under realistic dynamic conditions. The NEM-on-a-chip experiment indicated that the IBU-CS-β-GP formulation achieved a higher transport rate despite a lower delivered dose [114]. This formulation exhibited an 82.54 ± 0.2% permeation through the paracellular pathway, which might be attributed to chitosan’s permeation enhancing effect via interaction with epithelial tight junction proteins [109,118]. Crucially, the integrated electrochemical sensors enable rapid readouts of 28 s compared to high-performance liquid chromatography (HPLC, 7 min), highlighting the efficiency of on-chip sensing for high-throughput screening. Beyond transport kinetics, Gholizadeh et al. [109] also studied the in vitro interactions between aerosolized ibuprofen formulations and the human nasal epithelium under varying shear stress conditions. Sensors provide vital data on barrier integrity and cell health. By incorporating electrochemical sensors into the chip, researchers were able to measure the TEER values for RPMI 2650 cells exposed to IBU-CS-β-GP aerosol at 1.7 and 0.5 L/min and IBU-HBSS aerosol at 0.5 L/min. This innovative approach enables accurate observation of the impact of shear stress on cell viability and drug delivery efficiency. However, the reliability of electrochemical sensing in biological fluids is often hindered by non-specific interference from co-existing species. Consequently, current research focuses on engineering modified electrode interfaces that ensure selective analyte recognition while amplifying the detection signal [116].

In the area of toxicity assessment, an innovative in vitro model was developed by using live human nasal epithelial cells (hNECs) to study the toxicity of gaseous formaldehyde via airway delivery [119]. This method offers a more precise demonstration of the impact of formaldehyde toxicity on the human respiratory system. Moreover, advanced in vitro human nasal mucosa models hold promise for studying nasal diseases such as allergic rhinitis, chronic sinusitis, and nasal polyposis [120]. Integration of biosensors into these disease models may allow the continuous detection of inflammatory cytokines or metabolic stress markers, providing a dynamic profile of tissue response. All the models mentioned highlight the potential of the nasal mucosa-on-a-chip as a powerful tool for providing a deeper insight into nasal aerosol therapies and improving their validity in clinical applications [121].

Despite these promising advances, current OoC platforms still face challenges in generating mature tissue models with complete physiological characteristics, maintaining appropriate phenotypes across multiple cell types, and integrating multi-organ interactions. Studies have shown that other organs, such as the gut, can affect joint homeostasis [122]. This finding highlights organ-to-organ communication and points toward a research direction that considers the interplay among different parts of the human body. These constraints are relevant for intranasal CNS delivery studies, in which realistic modeling requires not only the nasal epithelium, but also vascular, BBB, brain, and immune-related components. First, complex systemic clearance processes—another key factor influencing nasal administration—are not yet fully reproducible in current OoC systems, as they depend on coordinated interactions across tissues and organs. Second, modeling the immune response of the human CNS remains challenging in vitro, because neuroinflammation depends on tightly regulated barrier properties and multicellular interactions within the neurovascular unit, including endothelial cells, astrocytes, pericytes, and microglia [123]. Third, the detection of low-abundance signaling molecules and the processing of complex biological samples remain difficult in microfluidic systems [113]. Material-related issues, such as the absorption of hydrophobic compounds by PDMS, may further complicate quantitative drug transport and clearance studies. Hence, continued advances in cell sourcing, tissue maturation, multi-organ coupling, and sensor performance will be necessary to improve the translational value of OoC-based intranasal drug evaluation.

## 5. Conclusions

This review has examined the emerging landscape of intranasal drug delivery for central nervous system disorders, encompassing the fundamental principles of nose-to-brain transport mechanisms, advanced nanotechnology-based delivery systems, microneedle platforms, and cutting-edge organ-on-chip evaluation technologies. In summary, the clinical realization of nose-to-brain delivery will depend on the synergistic integration of materials science, microfluidic evaluation, and patient-specific anatomical modeling.

### 5.1. Current Limitations and Clinical Translation Challenges

Despite advances in intranasal drug delivery technologies, several critical barriers impede clinical translation and widespread therapeutic application. As noted in Section 3, the most frequently cited challenges for nasal drug delivery include rapid mucociliary clearance and formulation stability, which limit drug residence time and therapeutic efficacy. The nasal cavity’s natural clearance mechanisms can remove administered formulations within 15–30 min, necessitating strategies to overcome this barrier [124]. Beyond formulation stability, the inherent physiological constraints of the nasal cavity also present fundamental challenges: the limited surface area of the olfactory region restricts drug absorption capacity. Additionally, inter-individual anatomical variations in nasal cavity structure and enzyme expression might create variability in drug absorption, complicating dose standardization.

From a formulation standpoint, achieving optimal physicochemical properties remains challenging. Nanocarrier systems must balance multiple competing requirements: particle size small enough for cellular uptake yet large enough to avoid rapid clearance, sufficient mucoadhesion without triggering inflammatory responses, and adequate drug loading capacity while maintaining stability [125,126]. Many nanocarrier studies still remain at the preclinical stage, with limited information on region-specific brain distribution, and non-standardized testing conditions. Moreover, the scalability of advanced formulations, involving complex surface modifications or multi-component systems, presents manufacturing hurdles. Microneedle-mediated intranasal delivery can enhance drug penetration through nasal mucosa while bypassing systemic circulation, offering direct CNS access for treating neurological disorders [58]. However, critical barriers to clinical translation persist, including manufacturing scalability issues, uneven drug distribution within nasal tissues, and the restricted therapeutic loading capacity dictated by current microneedle geometries [127]. Regarding drug evaluation, current organ-on-chip platforms still face critical limitations, including a reliance on immortalized cell lines that fail to recapitulate native nasal mucosa heterogeneity, lack of standardized protocols, and absence of integrated immune components. Addressing these problems necessitates further development in innovative key areas.

Regarding evaluation and regulatory gaps, the lack of standardized regulatory guidelines for intranasal CNS therapeutics remains a major bottleneck. Unlike well-established routes such as oral or intravenous administration, intranasal delivery lacks comprehensive regulatory guidelines [128]. Clinical translation of nose-to-brain therapeutics requires early consideration of safety, efficacy, and product quality rather than evaluation only at the approval stage. The FDA’s recent initiatives supporting alternative testing methods under the FDA Modernization Act 2.0 (2022) provide a framework for incorporating organ-on-chip data, yet specific guidance for nasal delivery applications remains under development. Although reformulated products may benefit from existing data through pathways such as FDA 505(b)(2), additional preclinical and clinical studies are still required when a new administration route is introduced [129]. Local safety evaluation requires histological assessment of the nasal mucosa and other exposed tissues (lung, bronchi), and potentially affected brain regions, highlighting the complexity of preclinical assessment [130]. Large-scale manufacturing must ensure batch-to-batch reproducibility, storage stability, and consistent device or particle performance. These remain challenging for complex nose-to-brain delivery platforms.

### 5.2. Future Perspectives

Beyond current applications in Alzheimer’s and Parkinson’s diseases, emerging opportunities include targeted therapy for brain tumors, stroke management, psychiatric disorders, and acute neurological emergencies where rapid CNS access is critical. Ultimately, the integration of personalized medicine principles will enable patient-specific formulations tailored to individual nasal anatomy, disease characteristics, and pharmacogenetic profiles.

Future innovations in formulation design will shift from passive carriers to smart, responsive systems. Nanotechnology innovations continue to drive progress in this field, with researchers developing increasingly sophisticated delivery platforms. Of particular note is the shift toward intelligent delivery systems capable of sensing and responding to their environment. Rather than relying solely on conventional mucoadhesion, they could respond to local nasal conditions such as pH and mucus properties, thereby improving spatial and temporal control over delivery. Meanwhile, the incorporation of bioinspired and biologically functionalized design strategies into nanocarrier systems may enhance barrier navigation, for example, through ligand-mediated targeting (e.g., WGA-functionalized nanoparticles and TAT-modified micelles) [37,38] and biomimetic surface engineering [131]. In terms of the diverse microneedle platforms, they collectively overcome traditional delivery limitations including first-pass metabolism, patient compliance issues, and dosage precision challenges. Future research might emphasize the optimization of nasal-specific microneedle geometries, the enhancement of mucoadhesive strategies, and the development of smart responsive systems capable of maximizing therapeutic efficacy while ensuring patient safety.

To bridge the gap between laboratory research and clinical translation, evaluation methodologies must evolve. Future organ-on-chip developments should focus on integrating additional physiological parameters including temperature gradients simulating nasal cavity thermal conditions, humidity control replicating the moist nasal environment, and mucus secretion dynamics with real-time viscosity monitoring. Capturing these dynamics necessitates a paradigm shift in on-chip sensing. Future platforms will likely transition from rigid, single-parameter electrodes to flexible, multi-modal sensing arrays capable of conforming to the 3D geometries of anatomical nasal replicas. Integrating immune components to model inflammatory responses will provide a more holistic view of safety. These platforms enable rapid drug screening, accelerating the development timeline for novel therapeutics. However, the most disruptive breakthrough lies in the integration of Artificial Intelligence (AI). Driven by the rapid development of neural networks, future nasal organ-on-chip platforms will likely integrate artificial intelligence and machine learning algorithms to enhance predictive capabilities for drug screening and personalized medicine applications [132]. The convergence of organ-on-chip technology with AI-assisted analysis systems will revolutionize drug evaluation by enabling real-time data interpretation and automated decision-making processes [133].

Next-generation nasal delivery systems are expected to incorporate patient-specific anatomical adjustments and advanced microfluidic models for more precise therapeutic outcomes [134]. Future directions include the development of patient-specific formulations based on individual nasal anatomy and disease characteristics, as well as combination therapies that leverage multiple delivery mechanisms simultaneously. The most significant breakthroughs will likely emerge from interdisciplinary collaborations bringing together materials scientists, neurobiologists, and computational experts. These advances are positioning intranasal delivery to become a mainstream therapeutic approach, especially for brain-targeted treatments.

## Data Availability

No new data were analyzed in this study.
